# Transport of Gold Nanoparticles by Vascular Endothelium from Different Human Tissues

**DOI:** 10.1371/journal.pone.0161610

**Published:** 2016-08-25

**Authors:** Radka Gromnicova, Mehmet Kaya, Ignacio A. Romero, Phil Williams, Simon Satchell, Basil Sharrack, David Male

**Affiliations:** 1 Department of Life, Health and Chemical Sciences, The Open University, Milton Keynes, United Kingdom; 2 Department of Physiology, Koc University School of Medicine, Istanbul, Turkey; 3 Midatech Pharma plc, Abingdon, United Kingdom; 4 School of Clinical Sciences, University of Bristol, Bristol, United Kingdom; 5 Department of Neurology, University of Sheffield, Sheffield, United Kingdom; University of Freiburg, GERMANY

## Abstract

The selective entry of nanoparticles into target tissues is the key factor which determines their tissue distribution. Entry is primarily controlled by microvascular endothelial cells, which have tissue-specific properties. This study investigated the cellular properties involved in selective transport of gold nanoparticles (<5 nm) coated with PEG-amine/galactose in two different human vascular endothelia. Kidney endothelium (ciGENC) showed higher uptake of these nanoparticles than brain endothelium (hCMEC/D3), reflecting their biodistribution in vivo. Nanoparticle uptake and subcellular localisation was quantified by transmission electron microscopy. The rate of internalisation was approximately 4x higher in kidney endothelium than brain endothelium. Vesicular endocytosis was approximately 4x greater than cytosolic uptake in both cell types, and endocytosis was blocked by metabolic inhibition, whereas cytosolic uptake was energy-independent. The cellular basis for the different rates of internalisation was investigated. Morphologically, both endothelia had similar profiles of vesicles and cell volumes. However, the rate of endocytosis was higher in kidney endothelium. Moreover, the glycocalyces of the endothelia differed, as determined by lectin-binding, and partial removal of the glycocalyx reduced nanoparticle uptake by kidney endothelium, but not brain endothelium. This study identifies tissue-specific properties of vascular endothelium that affects their interaction with nanoparticles and rate of transport.

## Introduction

Nanoparticles hold great potential in biomedicine; for diagnosis or as carriers of therapeutic agents to different tissues. However, a central problem is how the nanoparticles can be selectively delivered to the target tissue. Nanoparticles in the blood stream first interact with vascular endothelium before they may cross or pass the endothelial cells and enter the tissue. Vascular endothelium in different tissues has distinctive properties including its glycocalyx, surface receptors, intercellular junctions or rate of production of transport vesicles. These distinctive properties provide an opportunity to selectively target nanoparticles.

Most efforts have been directed towards understanding how the properties of the nanoparticle itself may change its interaction with the cell, i.e. whether it is taken up or becomes toxic for the cell. Several nanoparticle properties have been found to be crucial for this interaction; such as the size [1–7], shape [3,8,9], charge [7,10–13], ligand coating [14,15] as well as the proteins that may coat the nanoparticle once it comes into contact with serum [16].

Less attention has been paid to the cellular properties that influence nanoparticle transport. We have previously shown that glucose-coated gold nanoparticles (covalently bound glucose with a C2-linker) are transported across endothelium from brain, aorta or bone marrow at different rates [17]. Similarly, other studies have noted different rates of uptake by endothelia of different origin or epithelia [18–20]. Yet investigations to explain this phenomenon are lacking even though they may help to achieve tissue-selective targeting of nanoparticles.

Anatomical or physiological differences between different endothelia could explain differences in nanoparticle uptake and transport rates. In particular, the rate of uptake may be influenced by the plasma membrane properties of the cells and the binding of nanoparticles to cell surface glycoproteins and proteoglycans, as well as the cells’ capacity for vesicular transport. In each case, the first step in nanoparticle uptake or transcytosis is an interaction between the apical surface of the endothelial cell and the nanoparticle.

Potentially the first interaction with the endothelial cell will occur between the nanoparticles and components of the glycocalyx, which extends up to 500 nm from the cell surface [21]. Such interactions may depend on the cellular proteoglycans and the physical properties of the nanoparticles. This potential interaction is distinct from receptor-mediated binding which may be promoted by the attachment of specific targeting ligands to the nanoparticle [22], and depends on the nanoparticle reaching the receptor at the endothelial surface. Non-specific binding to the endothelium is a key element in absorptive endocytosis which precedes trans-endothelial transport. Endothelia from different tissues vary in their properties, including the glycocalyx, surface glycoproteins, receptors and vesicular transport systems, any of which could affect the rate of nanoparticle binding, internalisation and transcytosis.

Nanoparticles have been proposed as potential carriers of small drugs or biological agents into different tissues, for example, these can be gold nanoparticles coated with sugars, i.e. glyconanoparticles [23–25]. We have demonstrated that glucose-coated gold nanoparticles cross human brain endothelium *in vitro* and they can rapidly enter the brain of rats *in vivo*, following intra-vascular injection [17,26]. These studies also demonstrated differential uptake by endothelia from different vascular beds *in vitro*, and variable distribution to different tissues *in vivo*.

The current study investigates the cellular basis of these differences, with the ultimate aim of developing gold nanoparticles that are selective for different tissues, including brain microvessels. Specifically, we have examined cellular characteristics which may account for different rates of uptake of nanoparticles by human endothelial cells from brain or kidney. These properties include: (a) the surface glycocalyx; (b) differences in the rates of endocytosis (the sizes of vesicles and rate of internalisation); or (c) anatomical differences between the cells.

## Results and Discussion

### Characteristics of the gold nanoparticles

**Fig 1:**
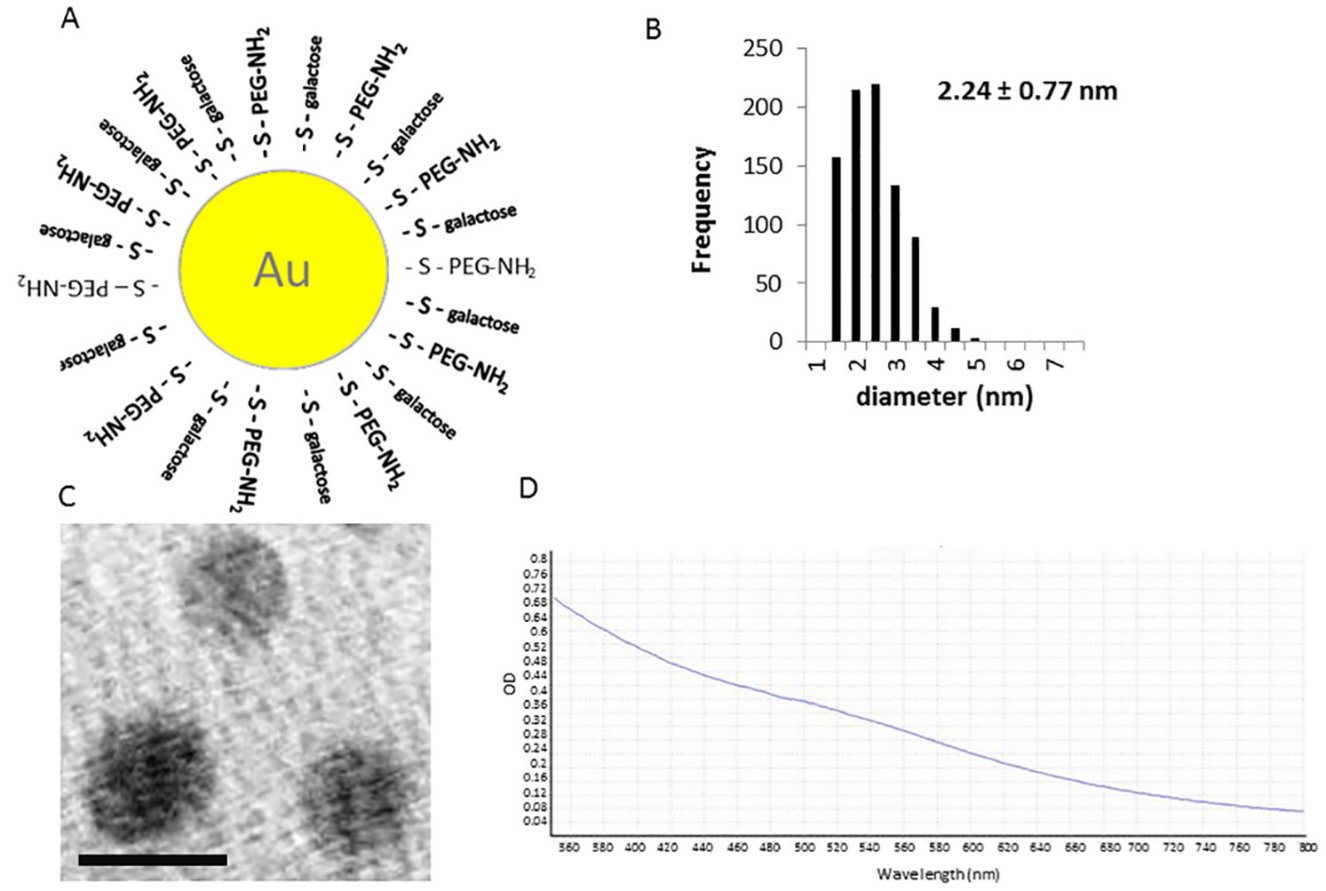
Characteristics of PEG-amine/galactose gold nanoparticles. (A) Schematic of nanoparticle organization. (B) Size distribution of nanoparticles (mean size ± s.d.). (C) High resolution electron microscopy of <4 nm nanoparticles (scale bar = 5 nm). (D) Absorbance spectrum of the nanoparticles.

The gold nanoparticles used in this study were coated with equal amounts of PEG-amine and a galactose derivative (PEG-amine/galactose), which were covalently bound to the gold core by a thiol linkage (Fig 1). These nanoparticles are similar to those used previously [15]: the galactose has a C2 linker to the gold core and the PEG-amine is EG_6_. Nanoparticles coated with PEG-amine/galactose were much more efficient in entering human brain endothelial cells in comparison with nanoparticles coated with galactose alone (Table 1). As the glycocalyx and plasma membrane of the endothelium are negatively charged, it has been proposed that cationic nanoparticles may be favoured in cell transport due to electrostatic interactions [27]. Our results confirm that the cationic PEG-amine/galactose nanoparticles are taken up much more efficiently than uncharged galactose-coated nanoparticles by the endothelium.

**Table 1:** Internalisation of gold nanoparticles by brain endothelial cells.

Nanoparticle coating	Cytosol	Vesicles
PEG-amine/galactose	0.26 ± 0.1*	2.07 ± 0.6
Galactose	0.007 ± 0.007	0.0003 ± 0.0001

* Values represent number of nanoparticles per micron of basal membrane from 85 nm TEM sections. Each value is the mean ± standard deviation of 3 independent experiments.

### Mechanisms of nanoparticle internalisation by endothelium

**Fig 2:**
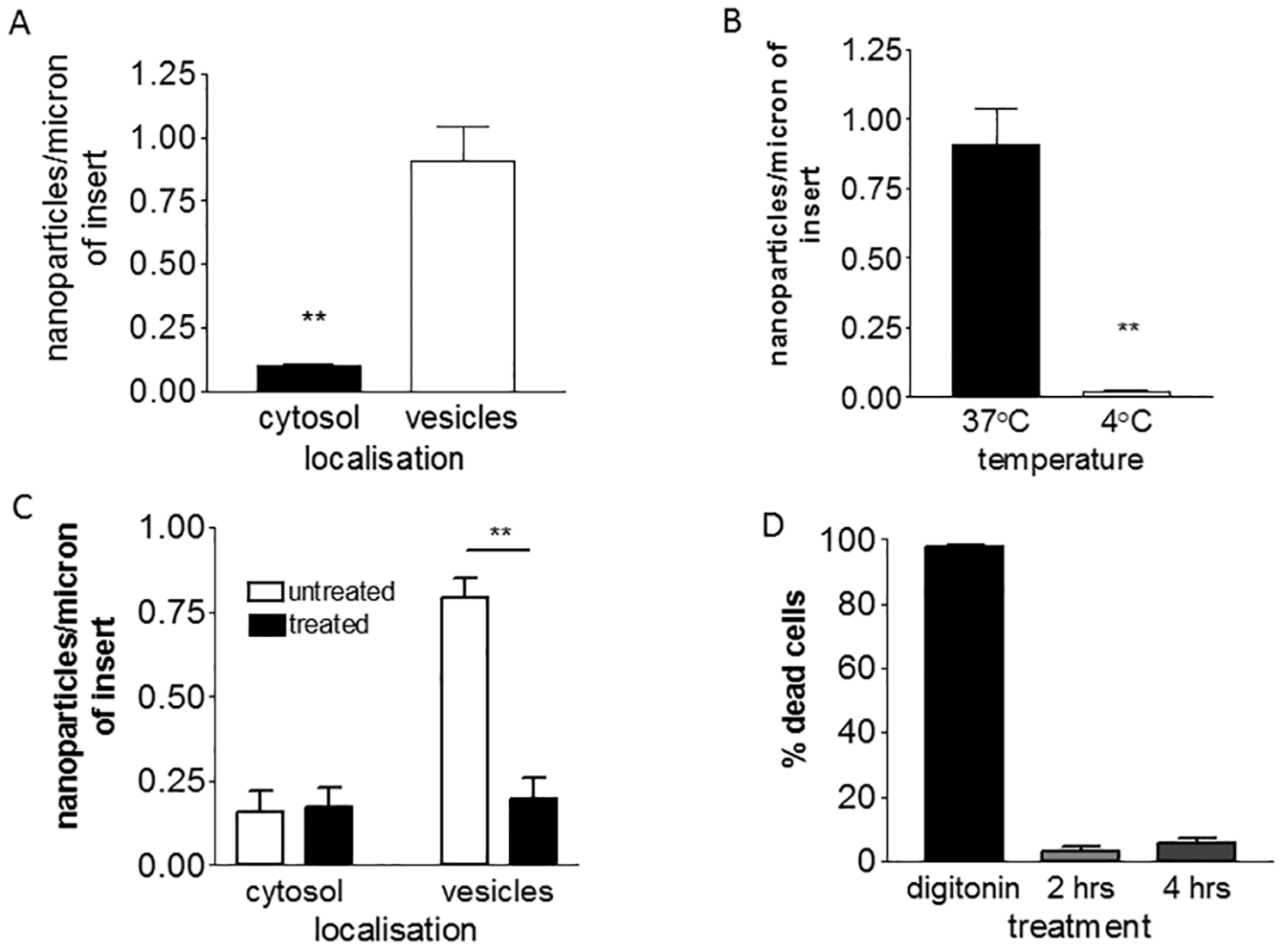
Uptake of PEG-amine/galactose gold nanoparticles by brain endothelial cells. (A) Localisation of nanoparticles at 3hrs (t-test, ** P<0.01). (B) Effect of temperature on nanoparticle uptake into vesicles at 3hrs (t-test, ** P<0.01). (C). Effect of inhibitors of active transport sodium azide/2-deoxy glucose on uptake of nanoparticles at 2 hrs (one-way ANOVA, Tukey’s post test ** P<0.001). (D) Cell membrane integrity/viability test after treatment with sodium azide/2-deoxy glucose, for 2 or 4hrs. Digitonin is a positive control for cell death. All bars show mean ± SEM and are all based on 3-independent experiments.

First, we investigated the mechanism by which PEG-amine/galactose nanoparticles are transported into the cells. Nanoparticles in different cellular compartments of brain endothelium were counted and it was noted that a high proportion localised in vesicles (Fig 2A). This implies that these nanoparticles primarily enter the cells by vesicular transport, an energy-dependent process. In order to confirm that transport into the brain endothelium was energy-dependent, the cells were incubated at 4°C (Fig 2B) or in the presence of sodium azide/2-deoxyglucose (Fig 2C). In both cases the numbers of vesicle-associated nanoparticles was significantly reduced. We considered the possibility that these inhibitors of cell metabolism could affect cell viability. To test this theory, cells were stained with propidium iodide, which only penetrates cells with damaged membranes: 95% of the cells were not stained (Fig 2D), hence we concluded that within the time-frame of the experiment, the cell viability was not affected.

In addition to nanoparticles that were localised in vesicles, some nanoparticles were present in the cytosol (Fig 2A). Immediate nanoparticle entry into the cytosol is thought to be energy-independent. It has been reported previously that gold nanoparticles (~2 nm) can enter the cytosol of cells by passively crossing the plasma membrane [28–31]. We have also previously reported that glucose-coated gold nanoparticles cross brain endothelium *in vitro* via the cytosolic route [17,32]. In accordance with these theories, cytosolic localisation of PEG-amine/galactose nanoparticles was not affected by sodium azide/2-deoxyglucose treatment (Fig 2C). On the other hand, incubation of the cells at 4°C reduced the number of cytosolic nanoparticles by about 50% (S1 Fig). This reduction is significant but smaller than the effect of low temperature on vesicular transport (Fig 2C). Passive uptake may be affected by membrane fluidity which is highly dependent on temperature—a less fluid membrane could decrease nanoparticle movement across the plasma membranes. Therefore, a change in membrane fluidity appears to be the most likely explanation for the reduction in nanoparticles localised in the cytosol at 4°C.

**Fig 3:**
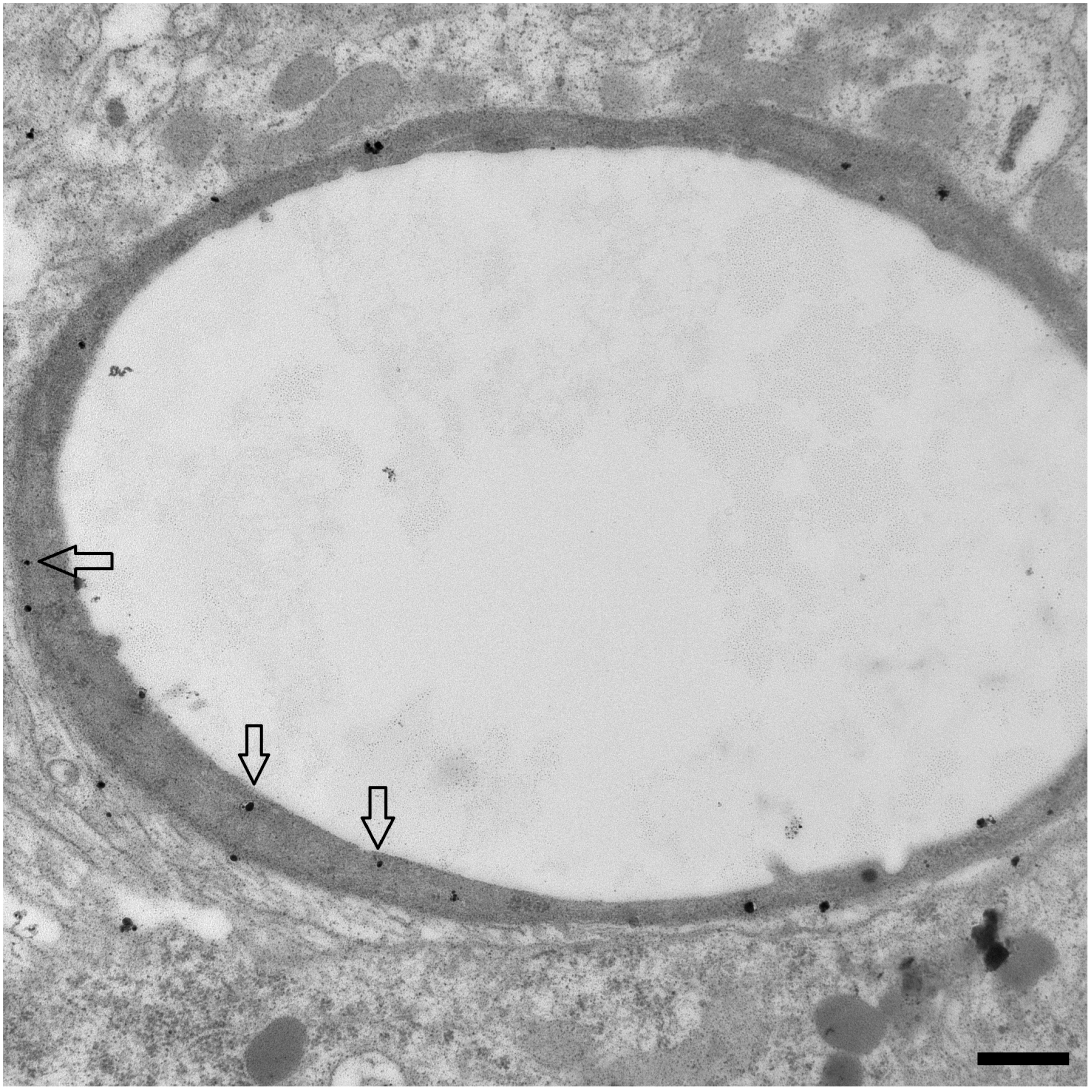
Location of PEG-amine/galactose nanoparticles in a brain vessel *in vivo*. Silver-enhanced electron micrograph of a microvessel of rat cerebral cortex, 10 minutes after intracarotid infusion of 50 μg (Au) of nanoparticles. Arrows indicate nanoparticles in the endothelium. Scale bar = 500 nm.

The mechanism of transport *in vitro* is correlated with observation of nanoparticle transcytosis *in vivo* (Fig 3). Following intracarotid infusion, nanoparticles are seen in brain microvascular endothelium, primarily in the cytosol. Within 10 minutes of infusion, the nanoparticles are also detected in cells of the brain parenchyma. Previous work has demonstrated that the nanoparticles move rapidly within the brain at speeds up to 1μm per minute.

To further analyse the mechanism of transport, brain endothelial cells were treated with inhibitors during the nanoparticle transport assay. In preliminary experiments nystatin, chlorpromazine, cytochalasin D and nocodazole were screened for their ability to inhibit the uptake of 70kDa FITC-dextran by brain endothelium. The doses of inhibitors were equivalent or greater than those previously reported [15,17] to inhibit endocytosis in non-brain endothelium, however, only nystatin and chlorpromazine were found to significantly inhibit FITC-dextran internalisation in the brain endothelium at the doses used, and in these conditions the viability of the hCMEC/D3 cells was slightly reduced (S1 Table). Nystatin inhibits caveolar uptake and chlorpromazine inhibits uptake into clathrin-coated vesicles. The results in the nanoparticle endocytosis-assay showed a reduction in vesicular uptake of up to 50% by both inhibitors, however, the difference was not significant due to high variability in cellular uptake (S2 Fig). Treatment with chlorpromazine significantly increased the number of nanoparticles in the cytoplasm and nucleus. However, we noted that the shape of the nucleus was irregular on treated cells. It is possible therefore that chlorpromazine is affecting the permeability of the plasma and nuclear membranes as well as cytoplasmic transport systems.

### Comparison of uptake by brain and kidney endothelium

We then investigated the rate of nanoparticle uptake in different endothelial cells. For this purpose we selected two human endothelial cell lines, from brain and kidney. The choice of cells was based on observations *in vivo* which showed that nanoparticles accumulated at highest levels in the kidney, and lowest in brain 10 minutes after intravascular infusion (S3 Fig). Initially, we hypothesised that the higher rate of accumulation in the kidney was related to structural differences such as fenestrations in kidney endothelium and tight junctions in brain endothelium. Alternatively, the difference could be due to surface properties of the endothelium or their rates of transcytosis.

**Fig 4:**
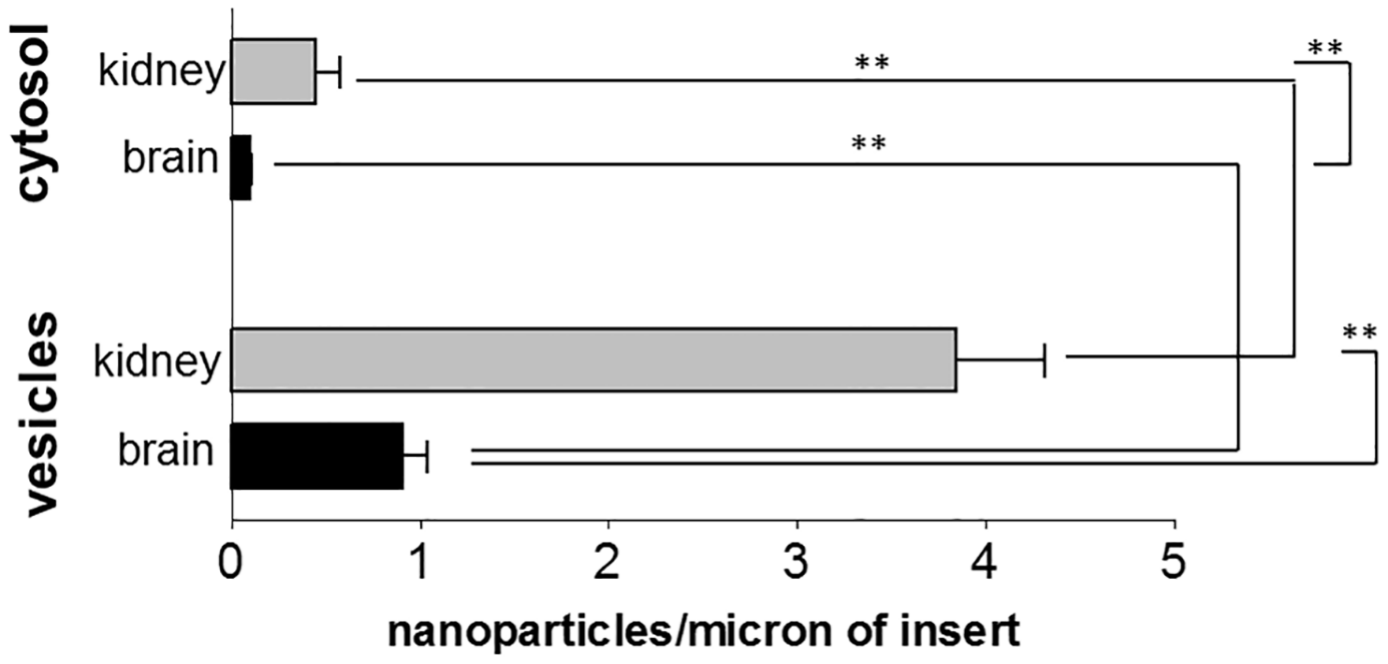
Uptake of nanoparticles by kidney or brain endothelial cells. Internalisation of PEG-amine/galactose gold nanoparticles into vesicles or cytosol of brain (hCMEC/D3) or kidney (ciGENC) endothelial cells at 3 hrs. Data are mean ± SEM of 3 independent experiments (ANOVA followed by Tukey’s multiple comparison test, P<0.01).

**Fig 5:**
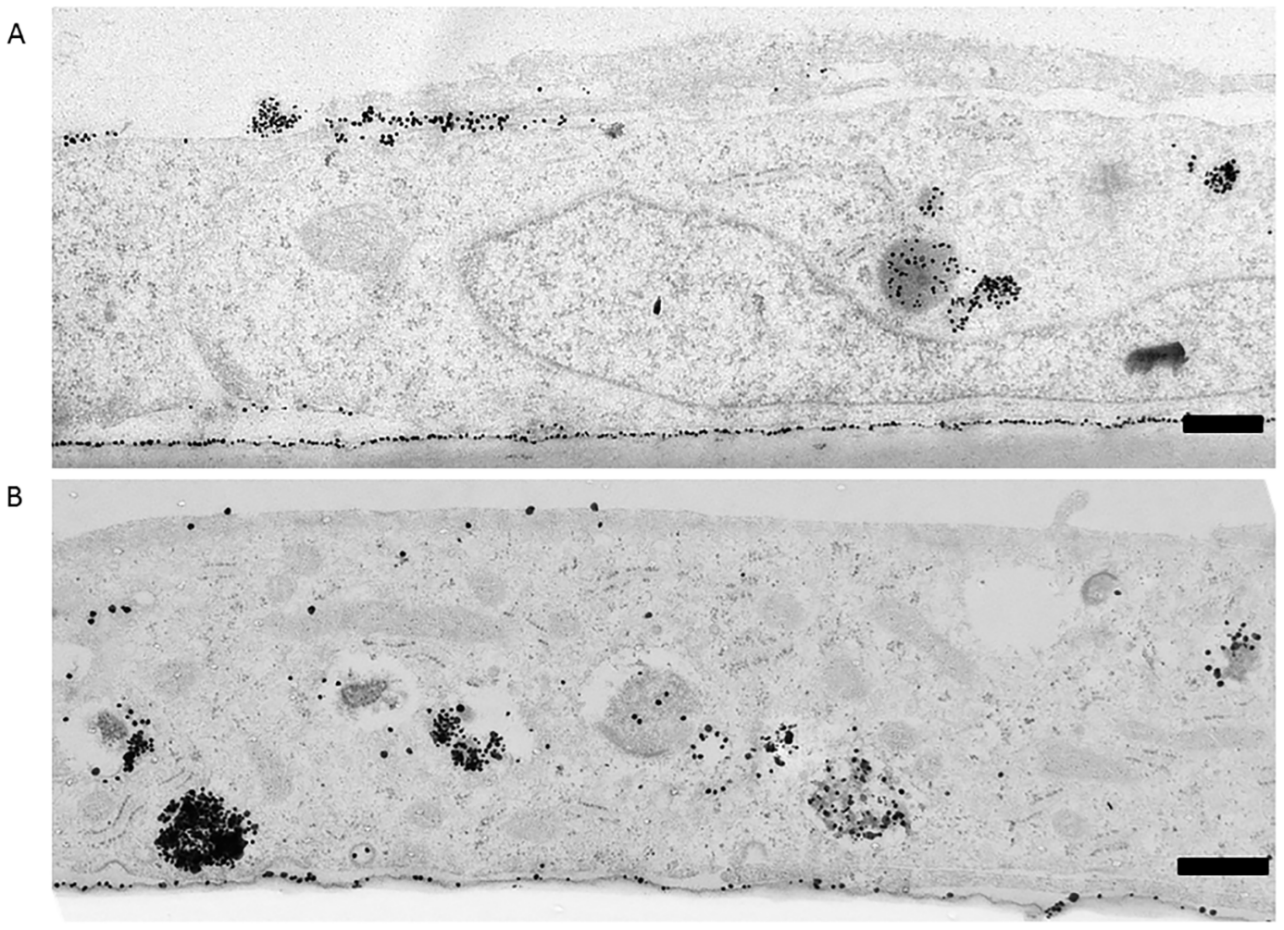
TEM of PEG-amine/galactose nanoparticles in endothelial cells. Silver-enhanced nanoparticles in endothelial cells, 3 hrs after their application to the apical (upper) cell surface. (A) Brain endothelial cells, hCMEC/D3. (B) Kidney endothelial cells, ciGENC. Scale bar = 0.5 μm.

The brain endothelial cell line hCMEC/D3 and the kidney glomerular endothelial line ciGENC are well characterised cells [33,34]. Both were immortalised by a similar process, involving transfection with SV40 large-T and both retain many of their tissue-specific properties. When we compared the uptake of PEG-amine/galactose nanoparticles in both endothelial cells, kidney endothelium showed higher nanoparticle uptake than brain endothelium, both into vesicles and cytosol (Figs 4 and 5). Both endothelial cell types showed approximately 4 times more nanoparticles in vesicles than in the cytosol. In both brain and kidney endothelium, there is an accumulation of nanoparticles on the transwell membrane, beneath the basal plasma membrane of the cells. These results imply that the nanoparticles have crossed the endothelium by cytosolic or vesicular transcytosis, and have been retained on the negatively charged filter.

### The role of the endothelial glycocalyx in nanoparticle internalisation

To explain the higher uptake of the nanoparticles by kidney endothelium we considered 3 possible mechanisms: (a) the surface properties of the endothelium are different so that the initial binding of the nanoparticles to the cells varies; (b) the rate of endocytosis is comparatively low in the brain endothelium; (c) morphological features of the cell lines, such as vesicle numbers or size of the cells may differ between the endothelia.

The initial interaction of a nanoparticle with the endothelium may occur at the glycocalyx. The glycocalyx is a brush-like structure that is located on the apical side of vascular endothelial cells. Its size can range from 0.5 microns to 11 microns deep [35–38] and it contains glycoproteins and proteoglycans which consist of glycosaminoglycans attached to core polypeptides. The glycocalyx is thought to be more fully developed *in vivo* than *in vitro* [39] and it is strongly negatively-charged, particularly on brain endothelium [40]. To investigate the role of the glycocalyx, we carried out a lectin-binding assay on the kidney and brain endothelium. Lectins bind to specific carbohydrate residues on the glycocalyx (Table 2) and they can be used to identify changes in glycocalyx composition [41]. They can also identify specialised domains on the plasma membrane [42].

**Table 2:** Glycan-binding properties of lectins on endothelium.

Lectin	Sugar specificity
Ulex Europaeus agglutinin (UEA)	-αL-fucose
Peanut agglutinin (PNA)	Gal-β(1–3)-GalNac
Wisteria Floribunda lectin (WFL)	GalNac
Wheat germ agglutinin (WGA)	GlcNac

**Fig 6:**
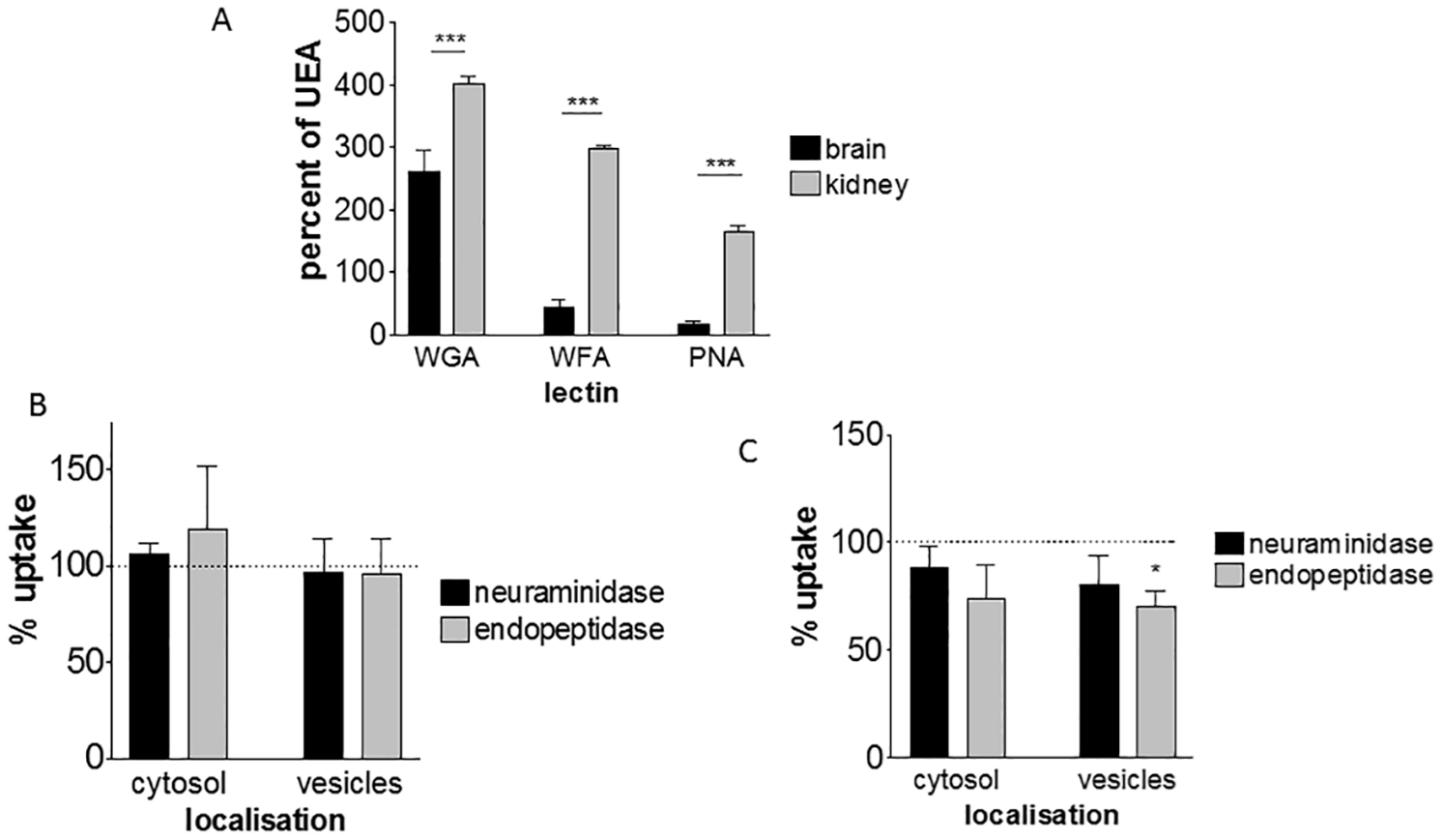
Effect of the endothelial glycocalyx on nanoparticle internalisation. (A) Binding profile of lectins WGA, WFL and PNA on brain and kidney endothelium. Binding was standardised as a percentage of UEA (standard endothelial marker), showing the mean ± SEM from 3 independent experiments (ANOVA and Tukey’s multiple comparison test, *** P<0.001). Effect of partial removal of glycocalyx (with neuraminidase or endopeptidase) on nanoparticle uptake into cytosol or vesicles, of brain endothelium (B) or kidney endothelium (C), compared with untreated cells (= 100%). (Data shown are mean ± SEM from 3 independent experiments, paired t-test, * P<0.05).

The lectin *Ulex Europaeus* agglutin (UEA) was used as a standard marker for endothelium [43]. The other three lectins were found to bind human endothelial cells in an initial screening of a panel of 23 lectins, of which 15 bound strongly to a panel of human endothelia (S2 Table). Wheat germ agglutinin (WGA) recognises N-acetyl glucosamine, including terminal sialic acid residues, whereas peanut agglutinin (PNA) and *Wisteria floribunda* lectin (WFL) primarily recognise internal residues. A schematic illustration of the proposed arrangement of the major lectin binding site, based on data in this study is shown in supplementary material (S4 Fig). Wheat germ agglutinin and peanut agglutinin have been shown to bind to microdomains of endothelium which are associated with transport vesicles (‘plasmalemmal vesicles’) [42]. We found that kidney endothelium exhibited a stronger binding profile to all lectins in comparison with brain endothelium (Fig 6A). This implies that the glycocalyx does indeed differ between the two cell types, and suggests that the kidney endothelium has higher numbers of domains associated with endocytosis.

To further analyse the role of the glycocalyx, enzymatic digestion was used to selectively remove carbohydrate residues. For partial removal of the glycocalyx we used neuraminidase and O-sialoglycoprotein endopeptidase (OSGEP). Neuraminidase removes sialic acid residues which contribute to the net negative charge of the glycocalyx, whereas the endopeptidase removes core polypeptides that have bound carbohydrate units. The lectin binding was changed by these enzymes (S5 Fig) confirming that elements of the glycocalyx, including the terminal sialic acid residues recognised by WGA, were removed. Chondroitinase and heparinase were also tested but they did not consistently affect lectin binding (data not shown). Neither neuraminidase nor endopeptidase treatment caused any change in the uptake of PEG-amine/galactose nanoparticles either into the cytosol or vesicles of brain endothelium (Fig 6B). However, neuraminidase produced a small reduction and endopeptidase a significant reduction in nanoparticle uptake by the kidney endothelium (Fig 6C).

**Fig 7:**
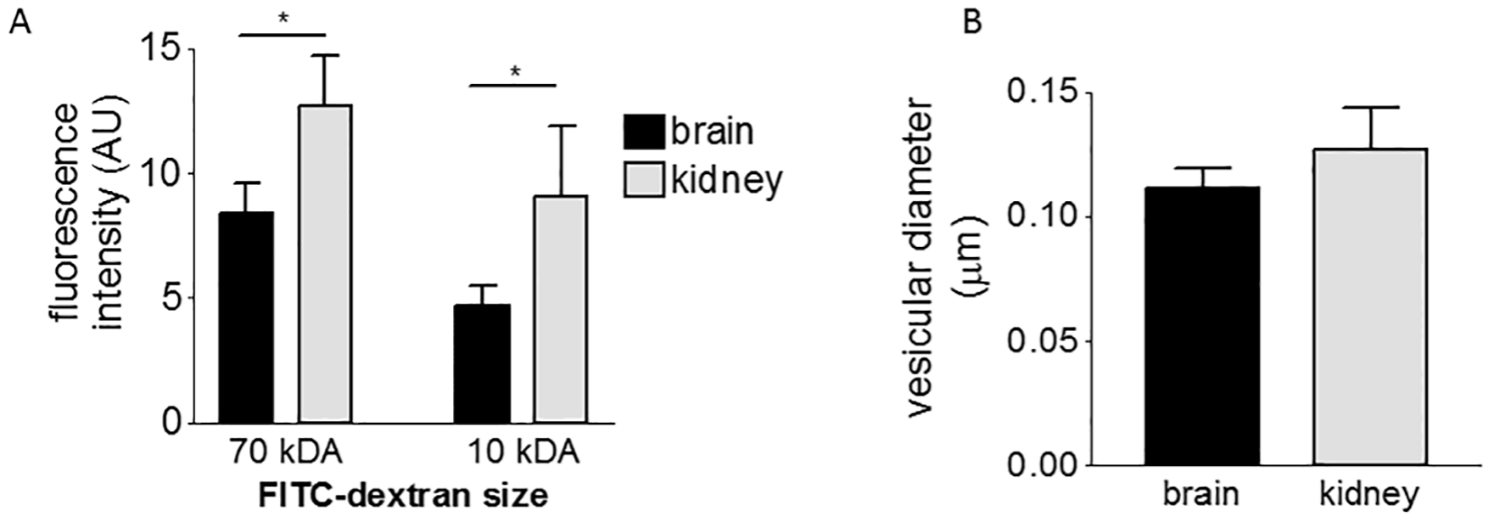
Comparison of the rate of endocytosis and vesicular size in endothelial cells. (A) Endocytosis of dextran measured by FACS, after 1hr incubation, comparing brain and kidney endothelial cells (mean ± SEM, 3 independent experiments, repeated-measures ANOVA with Bonferroni multiple comparison post test, * P< 0.05). (B) Diameter of vesicles in brain and kidney endothelial cells (mean ± SEM of 3 independent experiments, unpaired t-test, no-significant difference).

Initially, we hypothesised that removal of the negatively charged sialic acid residues by neuraminidase would increase nanoparticle uptake, by allowing the cationic nanoparticles to reach the plasma membrane more rapidly and thus enhance endocytosis. Therefore, the reduction in nanoparticle uptake following endopeptidase treatment was contrary to this hypothesis. One possible explanation is that the endopeptidase treatment disrupts microdomains (i.e. membrane subregions) which form the endocytotic vesicles, thus reducing endocytosis. The reduced nanoparticle uptake is only seen with kidney endothelium which has both higher levels of endocytosis as well as higher levels of lectin-binding sites than brain endothelium (Figs 6 and 7A).

The effect of the endothelial glycocalyx on nanoparticle uptake has not been investigated previously. The glycocalyx of erythrocytes has been shown to be crucial to the attachment of amphiphilic gold nanoparticles (around 2 nm core) to the cell membranes [44]. In this study, neuraminidase had a large effect on the nanoparticle attachment that was only slightly increased by treating with a combination of enzymes [44]. It is likely that fundamental differences in levels of endocytosis by erythrocytes and endothelium as well as the differences in the glycocalyx and use of different nanoparticles can explain why the endothelium takes up these nanoparticles so effectively by comparison with erythrocytes. It has been previously established that there is a 20 nm gap between each proteoglycan chain on the endothelial glycocalyx [45], which may allow smaller nanoparticles to freely pass through to the membrane surface.

### Differences in the rate of endocytosis, affecting nanoparticle uptake

We next investigated whether the different transport rates of the endothelia could be primarily due to differences in their overall rate of endocytosis, rather than the rate of nanoparticle binding. The rate of endocytosis can be investigated by loading cells with FITC-dextrans of different size. We selected FITC-dextran of 10 kDa and 70 kDa size, which correspond to a diameter of 4.4 nm and 12 nm, respectively [46]. Kidney endothelium showed higher fluorescence intensity when treated with both FITC-dextrans (Fig 7A) in comparison with brain endothelium. This may mean that the kidney endothelium has larger vesicles or has more vesicles because it is a larger cell. Firstly, we investigated vesicular radius and distribution of vesicles. However, we found that the mean vesicular radius was no different (Fig 7B) and both endothelia had a similar profile of vesicle sizes (S6 Fig). Secondly, as brain and kidney endothelial cells differ morphologically, we also investigated the cell volumes, but again we found that they were not significantly different (S7 Fig). Thus we concluded that the higher fluorescence intensity of FITC-dextran is due to a higher rate of endocytosis by kidney endothelium.

Taken together, these results show that the high rate of nanoparticle uptake by kidney endothelium may be explained by its higher rate of endocytosis. This may be correlated with its high level of surface lectin-binding domains, which are associated with endocytotic vesicles. *In vivo* endothelial cells show even more marked tissue-specific characteristics. Specifically, brain endothelium *in vivo* has very low numbers of transport vesicles (caveolae) by comparison with other endothelia. While other factors may affect transport into tissues, it is nonetheless the endothelium which controls the initial uptake of nanoparticles to each tissue and hence the endothelium is likely to be the critical cell type in determining the rate of nanoparticle entry into different tissues. This is particularly true for smaller nanoparticles which are not cleared by the mononuclear phagocyte system.

In summary, we have shown that galactose/PEG-amine-gold nanoparticles are actively taken up by brain and kidney endothelium primarily into vesicles. Smaller numbers enter the cells passively via the cytosolic route, crossing the plasma membrane. The rate of uptake was higher into kidney endothelial cells in comparison with brain endothelial cells. This is best explained by the higher overall rate of endocytosis by the kidney endothelium. The glycocalyx was also different between brain and kidney endothelium. Hence the possibility that the endothelial glycocalyx restricts nanoparticle access to the cell surface was investigated. However, partial removal of the glycocalyx actually reduced the rate of nanoparticle uptake by the kidney endothelium although it had no effect on nanoparticle uptake by brain endothelium. Thus, the glycocalyx does not appear to act as a barrier to these nanoparticles. Indeed disrupting the surface proteoglycans of kidney endothelium unexpectedly reduced nanoparticle internalisation. Consequently, it appears that the rate of endocytosis, rather than cell surface binding, is the main factor accounting for the different rates of nanoparticle internalisation by the different endothelia.

## Conclusions

Most research into the interactions of nanoparticles with cells has been concerned with how properties of the nanoparticles affect the cell. This study demonstrates how properties of the cell can influence nanoparticle uptake. We based our study on the observation of selective uptake of gold nanoparticles by human kidney endothelium in comparison with brain endothelium, since endothelial cells primarily determine the rate of entry of nanoparticles into different tissues. We concluded that gold nanoparticle uptake is correlated with the cell’s potential for active endocytosis. In addition, the surface glycocalyx affected active uptake of nanoparticles, but only for kidney endothelium. These findings in *vitro* reflect the tissue distribution of these small gold nanoparticles *in vivo*. Our work highlights the need to carry out detailed analysis of cell behaviour, in order to assess the potential use of nanoparticles as cell-selective or tissue-targeted carriers. Detailed analysis of both endocytosis and the interactions between nanoparticles and cell surface proteoglycans and glycoproteins may improve the design of such carriers. This information can help predict nanoparticle behaviour in the body.

## Materials and Methods

### Nanoparticle synthesis, characterisation and toxicity assessment

Gold nanoparticles were synthesized essentially as described previously [15]. Briefly, the organic ligand coating for this gold nanoparticle was comprised of two ligands: 1-thiohexaethylene glycol-17-ammonium acetate (Galchimia, Spain) and 2-thioethyl α-D-galactopyranoside as the disulphide (Galchimia, Spain). A 50:50 ligand starting ratio was used at the time of reduction of HAuCl_4_ with NaBH_4_ in 90% methanol and 10% water, with a 3-fold molar excess of total ligands to gold. After 2 hr shaking the nanoparticles were purified from unbound ligands and reactants by washing them 4x with excess water using a Millipore 10kDa ultra-centrifugation device. The nanoparticles were resuspended in water acidified to pH 6 with acetic acid and stored at 4°C prior to use.

The nanoparticle size was analysed using a transmission electron microscope (TEM). The nanoparticle solution was air-dried on a copper mesh grid slot and viewed on a JEM 1400 (JEOL, Japan) at an acceleration voltage of 80 kV and magnification of 80,000X. The images were analysed using ImageJ. The characteristic atomic mesh of the nanoparticles was observed on a high resolution TEM JEM 2100 (JEOL, Japan) at an acceleration voltage of 150 kV and magnification of 150,000X. The absorption spectrum of these nanoparticles was determined using a SPECTROstar Nano spectrophotometer (BMG Labtech, Germany).

The toxicity of PEG-amine/galactose gold nanoparticles was assessed on cells grown in 96-well plates. The nanoparticles were incubated with brain endothelial cells (hCMEC/D3) for 48 hrs at concentrations of up to 75 μg/ml at 37°C, with 4 replicates per treatment. The cells were then washed 2x with HBSS (Hank’s balanced salt solution, Sigma) and MTT [3-(4,5-dimethylthiazol-2-yl)-2,5-diphenyltetrazolium bromide] was added to the cultures for 3.5 hrs at 37°C. The solution was aspirated and 100 μl of DMSO (dimethylsulfoxide) was added to each well. The plate was then shaken for 10 min and the absorbance was read at 540 nm on a plate reader OPTIMA FluoStar (S8 Fig).

### Cell cultures

The immortalized brain endothelial cell line, hCMEC/D3 [47], was propagated in EBM-2 MV medium with 2.5% foetal bovine serum, 0.025% VEGF, IGF and EGF; 0.1% bFGF, gentamycin, amphotericin B and ascorbic acid; 0.04% hydrocortisone. The medium was changed every 2–3 days and the cells cultured between passages 25 and 33. The cells were kept at 37°C in humidified atmosphere with 5% CO_2_.

The conditionally immortalized glomerular endothelial cells (ciGENC) were supplied by Simon Satchell [33]. They were propagated in EBM-2 MV supplemented according to the manufacturer’s protocol, with the exception of growth factor VEGF, which was omitted from the medium. The medium was changed every 2–3 days. The propagating cultures were grown at 33°C. For experiments the cultures were kept at 33°C until they were 90% confluent, then they were kept at 37°C for 3–5 days in order to develop kidney glomerular endothelial phenotype before they were used. In each case, the cells were kept in a humidified atmosphere with 5% CO_2_. In all assays the different endothelial cells were cultured in identical conditions.

Cell viability following antibiotic treatment was measured by trypan blue exclusion. The hCMEC/D3 cells (10^5^) were stained with 0.04% trypan blue in PBS and the percentage of stained (non-viable) cells counted. The background percentage of non-viable cells in untreated cultures was subtracted from the values stated.

To assess the active uptake involvement in transport of PEG-amine/galactose gold nanoparticles, the hCMEC/D3 cells were cultured for 3 hrs at 4°C or for 2 hrs with inhibitors of active metabolism NaN_3_ (10 mM) + 2-deoxyglucose (50 mM) and processed for TEM (see below). The possible cytotoxic effect of NaN_3_ + 2-deoxyglucose on cells was assessed by staining cells with propidium iodide after 2 or 4 hr treatment. Digitonin (30 μg/ml for 15 min) was used as a positive control for cell death, and fluorescence intensity was measured on a Facscan, channel FL2 set at 400V gain: 10,000 events were analysed from the gated cell population.

### Transmission electron microscopy to quantify the localisation and number of nanoparticles in cells

The rate of transport of PEG-amine/galactose gold nanoparticles into and across cells was assessed by quantification of the number of nanoparticles in cells by transmission electron microscopy (TEM). The cells were grown on transwell inserts (1 cm^2^, Corning Costar), the nanoparticles (8 μg/ml) were applied to the top chamber in EBM-2 MV medium supplemented with 2.5% FBS. The time of incubation was 2–3 hrs. After the incubation, both chambers were washed 3x in HBSS. The cells were fixed in 2.5% glutaraldehyde for 1 hr at room temperature. The fixative was removed, both chambers washed 3x in PBS (phosphate buffer saline) and stored in phosphate buffer at 4°C.

The samples were processed for TEM (all incubations were performed at room temperature and all solutions were applied to both the insert and the bottom chamber). Firstly, the cells were permeabilized in 0.01% Triton x100 for 15 minutes on a rocker. The inserts were washed 3x in PB (phosphate buffer) and silver enhanced. Silver enhancement (Aurion) was prepared according to the manufacturer’s protocol and applied to the insert for 45 min. 3 washes in distilled water followed. The cells were then treated with 1% osmium tetroxide for 30 min. The insert was washed 3x in PB and removed from the well; the membrane was cut in 2 pieces of about 2 x 3 mm each. The membranes were then gradually dehydrated in ethanol: 30% and 50% for 5 min, 70% and 90% for 10 min, 100% for 10 min twice, 100% with molecular sieve for 10 min. The membranes were incubated in a 50:50 mixture of 100% ethanol and Epon resin overnight and then in fresh resin twice for 2 hrs. The embedding took place at 60°C for 48 hrs. Resin blocks were microsectioned to 80 nm sections using a Diamond knife (Diatome, Switzerland). The sections were collected onto pioloform film-covered copper grids. They were stained in 1% uranyl acetate (30 min) followed by lead citrate (10 min). Sections were viewed on a JEM 1010 (Jeol, Japan) at an acceleration voltage of 80 kV. The cells were observed at magnification of 20,000x which allowed sufficient magnification to view the nanoparticles. The nanoparticles were counted and sorted into categories “vesicles” or “cytosol” during viewing on the electron microscope. The length of insert which was viewed (usually around 1000 microns in length) was measured with low magnification of 150X and the number of nanoparticles was recalculated to obtain number of nanoparticles per micron of insert.

### Characterisation and modulation of the endothelial glycocalyx

In order to characterise the endothelial glycocalyx, cells were stained using biotinylated lectins. hCMEC/D3 cells or ciGENC cells were cultured in a 96 well plate for 3 and 4 days respectively. The cells were washed twice in HBSS, fixed in 0.1% glutaraldehyde (in PBS) for 15 min. They were washed twice in PBS, blocked in 0.05M Tris/HCl for 20 min and washed 3x in wash buffer (0.05% Tween20 in PBS). Lectins were added for 1 hr at concentrations: *Ulex Europaeus* agglutinin 10 μg/ml, Wheat germ agglutinin 5 μg/ml, Peanut agglutinin 20 μg/ml, *Wisteria floribunda* lectin 20 μg/ml; all prepared in diluent (5 mg/ml BSA and 0.01% Tween20 in PBS). After the incubation, the cells were washed in wash buffer 3x. Streptavidin peroxidase (1:700 in 5 mg/ml BSA and 0.01% Tween20 in PBS) was added to the cells for 1hr, then washed in wash buffer 3X and once in PBS. The chromogen solution containing 100 μg/ml tetramethylbenzidine in 0.1M sodium acetate/citric acid was prepared and hydrogen peroxide added to a final concentration of 1/3000. This solution was added to the cells for 10–15 min resulting in blue colour; the reaction was stopped by adding 20 μl of 30% H_2_SO_4_ per well. The absorbance was read at 450 nm on a plate reader OPTIMA FluoSTAR. Data are expressed as mean absorbance from replicate wells, of a representative experiment, which had been repeated 3 times.

Endothelial cells were also treated with enzymes for 2 hrs, to remove parts of the glycocalyx. The enzymes used were 50 mU/ml neuraminidase or 25 μg/ml endopeptidase. After the treatment, the cells were washed twice in HBSS and either stained with lectins or treated with nanoparticles (as described above).

### Analysis of rate of endocytosis

The cells were treated with FITC-dextran of 70 kDa or 10 kDa in order to test the rate of endocytosis. The cells were grown in 12-well plates and FITC-dextran was applied at 0.2 mg/ml for 1 hr. The cells were washed with ice-cold HBSS 5x over 5 min; they were trypsinized, collected and washed 2x in HBSS + 0.1 mg/ml BSA. Then they were resuspended in PBS and analysed using flow cytometry (BD FacScan, channel FL1, 460#x2014;500V: 10,000 events analysed from the gated cell population). Data are expressed as the mean of the median fluorescence values from 3 independent experiments.

### Analysis of vesicular diameter and cell volume

TEM sections of hCMEC/D3 and ciGENC cells were used to analyse vesicular diameter and cell volume. Each section corresponded to a well with a single treatment. As each treatment had 3 wells, one was randomly picked for this analysis and 3 independent experiments were analysed per cell type, resulting in 3 sections analysed altogether. At 20,000x magnification on JEM 1010 (Jeol, Japan), about 100 vesicular diameters were measured per section in cells that were randomly picked. The data set was analysed with Microsoft Office Excel 2010 and GraphPad Prism. Similarly, to obtain volumes of brain cells in comparison to kidney cells, 3 independent experiments were analysed for each cell type. At magnification of 2,000X on JEM 1010 (Jeol, Japan) 10 images per treatment were taken randomly. The cell area was measured using ImageJ. The data were analysed as a distribution histogram and means of each independent experiment were calculated and analysed by GraphPad Prism.

### In vivo study of nanoparticle tissue-distribution

For the animal study, 4 adult male Wistar albino rats weighing 250–300 g were used (Vivarium from Bogazici University, Istanbul, Turkey) for testing nanoparticle distribution.

This work was approved by the Local Ethics Committee for Animal Experimentation of Istanbul University (2014/06).

The animals were anaesthetized with chloral hydrate (360 mg/kg, i.p.) and locally anaesthetized with bupivacaine hydrochloride. After the incision in the neck, the left common internal and external carotid arteries were exposed. The external carotid was ligated and the artery was retrogradely catheterized. Each animal was infused with warm 50 μg (100 μl) of nanoparticle solution (prepared in water) through the exposed external carotid artery over 2 min using an infusion pump (Harvard Apparatus, Infusion/Withdrawal pump, Model No: 901A). The nanoparticles were allowed to circulate for 10 min and the animals were administered with sodium pentothal (50 mg/kg, i.p.) and chloral hydrate (100 mg/kg, i.p.) before perfusion/fixation procedure. The animals were perfused transcardially with 0.9% saline (75 ml), and fixed with 200 ml of 2.5% glutaraldehyde and 2% paraformaldehyde in 0.1 M PB for 15 min. The brain, liver, kidney and lung were collected and stored for the analysis.

To analyse the amount of gold in the organs, ICP-MS (inductively-coupled plasma massed spectrometry) was used. Boiling HNO_3_ was used to digest brain, liver, kidney and lung for 5–10 min. After diluting the samples to 5 ml volume, they were run in 1% HCl with Iridium standard (10 ng/ml) on NexION ICP-MS (PerkinElmer, USA). The ICP-MS produced data on the amount of gold per gram of tissue. In order to account for the differences in individual size (body weight) of the animals, these values were compensated according to the weight of each animal, resulting in relative levels of gold in each tissue.

## Supporting Information

S1 Fig Uptake of cytosolic nanoparticles at 4°C compared with 37°C (= 100%). Data show mean ± SEM of 3 independent experiments. Unpaired t-test, ** P< 0.01.
(TIF)

S2 Fig Inhibition of nanoparticle active transport by antibiotics. Brain endothelial cells (hCMEC/D3) were treated with 30μg/ml chlorpromazine or 50μg/ml nystatin during a nanoparticle uptake assay for 1hr. Results show mean ± SEM of 2-independent experiments, comparing treated and untreated cells. * P <0.05.
(TIF)

S3 Fig In vivo uptake of PEG-amine/galactose—gold nanoparticles in rat tissues following infusion into the carotid artery. Nanoparticles were allowed to circulate for 10 minutes before perfusion to remove nanoparticles from the vasculature. Gold was measured by ICP-mass spectrometry. Values are mean ± SEM of 4 animals.
(TIF)

S4 Fig A scheme for lectin binding sites on endothelial cells. PNA = peanut agglutinin, WFL = Wisteria floribunda lectin, WGA = Wheat germ agglutinin. Neuraminidase removes the terminal sialic acid to reduce binding of WGA and enhance binding of PNA and WFL (See S5 Fig).
(TIF)

S5 Fig Test of enzymatic removal of glycocalyx on kidney and brain endothelial cells. Binding of lectin PNA (A) and (C) to glycocalyx of kidney (A) and brain endothelial cells (C) after enzymatic removal with endopeptidase (endo) or neuraminidase (neura). Binding of lectin WGA (B) and (D) to kidney (B) and brain endothelial cells (D) after enzymatic removal with endopeptidase and neuraminidase. ANOVA Tukey’s multiple comparison *P<0.05, **P<0.01, ***P<0.001. Data shown as mean ± SEM of 3 independent experiments.
(TIF)

S6 Fig A profile analysis of vesicular diameter for brain (A) and kidney (B) endothelial cells. (TIF)

S7 Fig Comparison of cell volume area of brain (hCEMC/D3) and kidney (ciGENC) endothelial cells. The cell volume area was analysed from sections viewed on the electron microscope. 3 independent experiments, data shown as mean +-SEM, t-test non-significant.
(TIF)

S8 Fig *In vitro* toxicity of gold nanoparticles on brain endothelial cells (hCMEC/D3). MTT assay of nanoparticles coated with PEG-amine/galactose of varying concentrations at 48 hrs exposure to the cells (n = 3). Digitonin treatment is a control of cell death. Data shown as mean ±SEM.
(TIF)

S1 Table Viability of hCMEC/D3 cells treated with antibiotics. Viability was measured by trypan blue staining. Results are mean ±SD from 3 independent experiments with duplicate determinations.
(DOCX)

S2 Table Initial screen of lectin-binding to human endothelial cells. Binding of biotinylated lectins (10μg/ml) was compared with the level of binding of 5 μg/ml antibody to MHC class-I (standard). Results are from 3 experiments and are expressed as the binding range for each lectin, where— = no detectable binding, 1 = <25%, 2 = 25%-75%, 3 = 75%-125%, 4 = 125%-175% and 5 = >175% of the MHC class-I. Lectins used were: ConA, concanavalin-A; DBA, Dolichus biflorus agglutinin; DSL, Daturum stramonium lectin; ECL, Erythina crystagalli lectin; GSL, Griffonia (Bandeiraea) simplificifolia lectins I, II and isolectin B4; Jacalin; LCA, Lens culinaris agglutinin; LEL, Lycopersicon esculentum (tomato) lectin; PHA-E, Phaseus vulgaris erythroagglutinin; PHE-L, Phaseus vulgaris leucoagglutinin; PNA, peanut agglutinin; PSA, Pisum sativum agglutinin; RCA1, Ricinus communis agglutinin; SBA, Soybean agglutinin; SJA, Sophora japonica agglutinin; STL, Solanum tubersosum (potato) lectin; UEA I, Ulex europaeus agglutin I; VVL, Vicia villosa lectin; WFL, Wisteria floribunda lectin; WGA, Wheat germ agglutinin; sWGA, succinylated wheat germ agglutinin. Human endothelial cells were prepared as described (Hillyer P and Male DK (2005) Expression of chemokines on the surface of different human endothelia. Immunol. Cell Biol. **83**, 375–382) and those used were: BMEC, Bone marrow endothelial cells; SVEC, saphenous vein endothelial cells; HUVEC, human umbilical vein endothelial cells; DMVEC, dermal microvascular endothelial cells; LMVEC, lung microvascular endothelial cells.
(DOCX)
