# Supplementary figures and images for: Transport of Gold Nanoparticles by Vascular Endothelium from Different Human Tissues
Source: PLoS One. 2016 Aug 25;11(8):e0161610. doi: 10.1371/journal.pone.0161610 (PMC4999129; doi:10.1371/journal.pone.0161610)

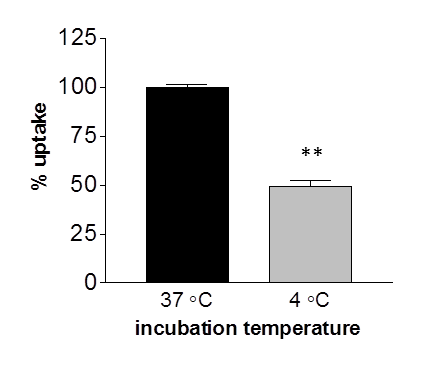

Supplement: S1 Fig — Data show mean ± SEM of 3 independent experiments. Unpaired t-test, ** P< 0.01. (TIF) [file pone.0161610.s001.TIF]

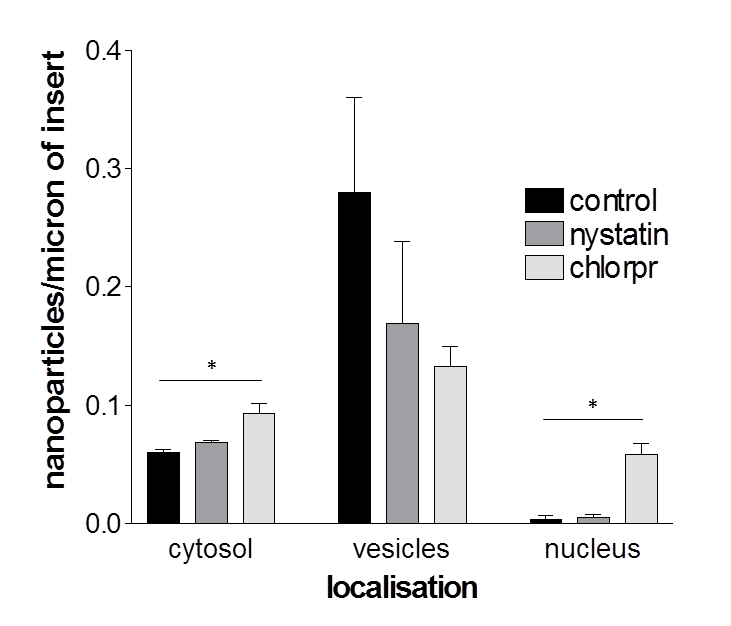

Supplement: S2 Fig — Brain endothelial cells (hCMEC/D3) were treated with 30μg/ml chlorpromazine or 50μg/ml nystatin during a nanoparticle uptake assay for 1hr. Results show mean ± SEM of 2-independent experiments, comparing treated and untreated cells. * P <0.05. (TIF) [file pone.0161610.s002.tif]

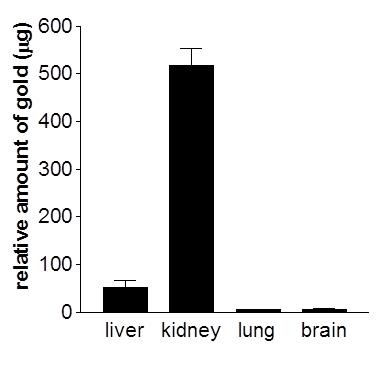

Supplement: S3 Fig — Nanoparticles were allowed to circulate for 10 minutes before perfusion to remove nanoparticles from the vasculature. Gold was measured by ICP-mass spectrometry. Values are mean ± SEM of 4 animals. (TIF) [file pone.0161610.s003.TIF]

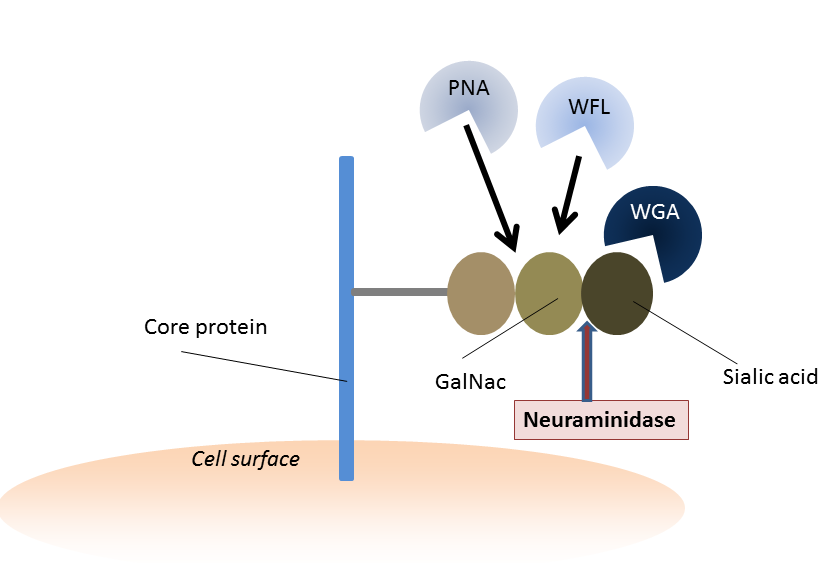

Supplement: S4 Fig — PNA = peanut agglutinin, WFL = Wisteria floribunda lectin, WGA = Wheat germ agglutinin. Neuraminidase removes the terminal sialic acid to reduce binding of WGA and enhance binding of PNA and WFL (See S5 Fig). (TIF) [file pone.0161610.s004.TIF]

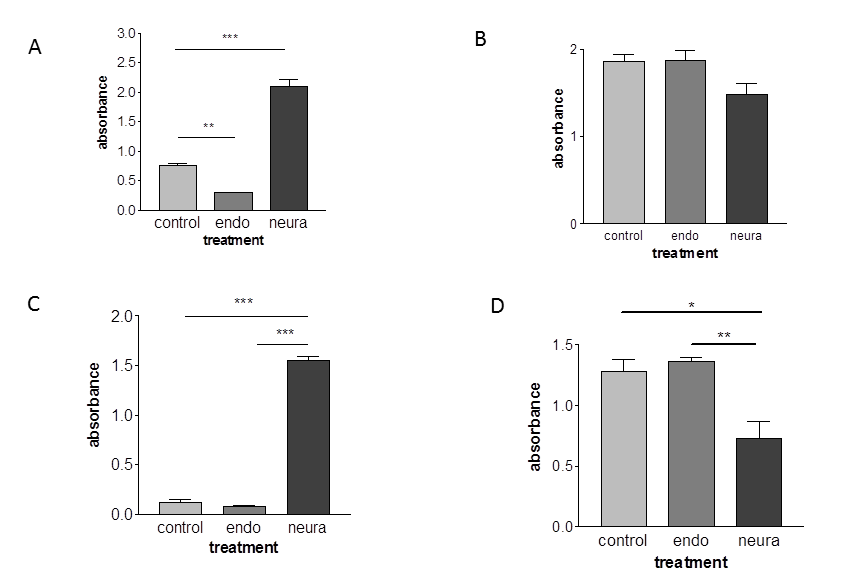

Supplement: S5 Fig — Binding of lectin PNA (A) and (C) to glycocalyx of kidney (A) and brain endothelial cells (C) after enzymatic removal with endopeptidase (endo) or neuraminidase (neura). Binding of lectin WGA (B) and (D) to kidney (B) and brain endothelial cells (D) after enzymatic removal with endopeptidase and neuraminidase. ANOVA Tukey’s multiple comparison *P<0.05, **P<0.01, ***P<0.001. Data shown as mean ± SEM of 3 independent experiments. (TIF) [file pone.0161610.s005.TIF]

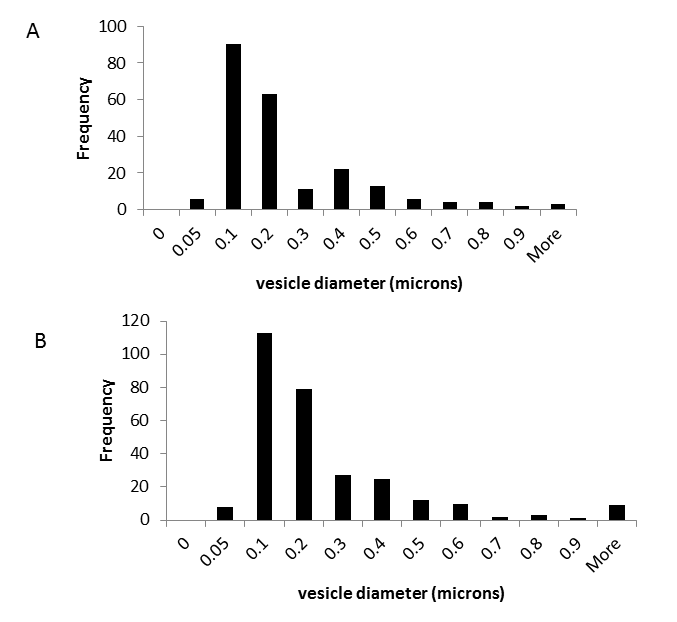

Supplement: S6 Fig — (TIF) [file pone.0161610.s006.TIF]

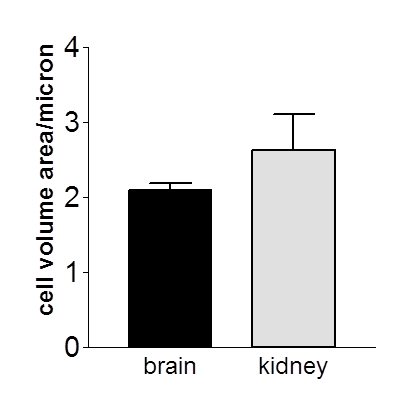

Supplement: S7 Fig — The cell volume area was analysed from sections viewed on the electron microscope. 3 independent experiments, data shown as mean +-SEM, t-test non-significant. (TIF) [file pone.0161610.s007.TIF]

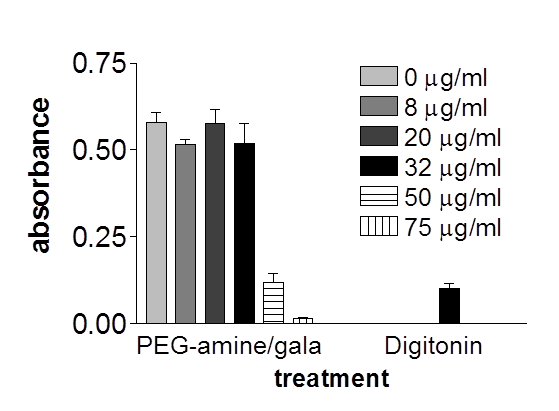

Supplement: S8 Fig — MTT assay of nanoparticles coated with PEG-amine/galactose of varying concentrations at 48 hrs exposure to the cells (n = 3). Digitonin treatment is a control of cell death. Data shown as mean ±SEM. (TIF) [file pone.0161610.s008.TIF]
