# Supplementary material for: Transport of Gold Nanoparticles by Vascular Endothelium from Different Human Tissues
Source: PLoS One. 2016 Aug 25;11(8):e0161610. doi: 10.1371/journal.pone.0161610 (PMC4999129; doi:10.1371/journal.pone.0161610)
Supplement: S1 Table — Viability was measured by trypan blue staining. Results are mean ±SD from 3 independent experiments with duplicate determinations. (DOCX) [file pone.0161610.s009.docx]

**Supplementary Table 2.**  **Viability of hCMEC/D3 cells treated with antibiotics.**

| **Treatment** | **30 minutes** | **60 minutes** |
| --- | --- | --- |
| **50µg/ml Nystatin** | 81.9 ± 27.5% | 86.4 ± 7.3% |
| **30µg/ml Chlorpromazine** | 67.7 ± 14.7% | 76.8± 16.2% |

* Viability was measured by trypan blue staining. Results are mean ±SD from 3 independent experiments with duplicate determinations.
