# Supplementary material for: Transport of Gold Nanoparticles by Vascular Endothelium from Different Human Tissues
Source: PLoS One. 2016 Aug 25;11(8):e0161610. doi: 10.1371/journal.pone.0161610 (PMC4999129; doi:10.1371/journal.pone.0161610)
Supplement: S2 Table — Binding of biotinylated lectins (10μg/ml) was compared with the level of binding of 5 μg/ml antibody to MHC class-I (standard). Results are from 3 experiments and are expressed as the binding range for each lectin, where— = no detectable binding, 1 = <25%, 2 = 25%-75%, 3 = 75%-125%, 4 = 125%-175% and 5 = >175% of the MHC class-I. Lectins used were: ConA, concanavalin-A; DBA, Dolichus biflorus agglutinin; DSL, Daturum stramonium lectin; ECL, Erythina crystagalli lectin; GSL, Griffonia (Bandeiraea) simplificifolia lectins I, II and isolectin B4; Jacalin; LCA, Lens culinaris agglutinin; LEL, Lycopersicon esculentum (tomato) lectin; PHA-E, Phaseus vulgaris erythroagglutinin; PHE-L, Phaseus vulgaris leucoagglutinin; PNA, peanut agglutinin; PSA, Pisum sativum agglutinin; RCA1, Ricinus communis agglutinin; SBA, Soybean agglutinin; SJA, Sophora japonica agglutinin; STL, Solanum tubersosum (potato) lectin; UEA I, Ulex europaeus agglutin I; VVL, Vicia villosa lectin; WFL, Wisteria floribunda lectin; WGA, Wheat germ agglutinin; sWGA, succinylated wheat germ agglutinin. Human endothelial cells were prepared as described (Hillyer P and Male DK (2005) Expression of chemokines on the surface of different human endothelia. Immunol. Cell Biol. 83, 375–382) and those used were: BMEC, Bone marrow endothelial cells; SVEC, saphenous vein endothelial cells; HUVEC, human umbilical vein endothelial cells; DMVEC, dermal microvascular endothelial cells; LMVEC, lung microvascular endothelial cells. (DOCX) [file pone.0161610.s010.docx]

**Supplementary Table 1. Initial screen of lectin-binding to human endothelial cells**

| **Lectin*** | **Endothelial cell type^¶^** | | | | |
| --- | --- | --- | --- | --- | --- |
|  | **BMEC** | **SVEC** | **HUVEC** | **DMVEC** | **LMVEC** |
| **ConA** | **5** | **4** | **5** | **4-5** | **5** |
| **DBA** | **1** | **1** | **1** | **1** | **1** |
| **DSL** | **4-5** | **3** | **4** | **3-4** | **4** |
| **ECL** | **3-4** | **3** | **3-4** | **3** | **3-4** |
| **GSL I** | **2** | **2** | **1** | **2** | **2** |
| **GSL II** | **2** | **2** | **1-2** | **1** | **1** |
| **GSL IB4** | **1-2** | **2** | **1** | **1** | **1** |
| **Jacalin** | **2-5** | **4** | **1-2** | **2** | **1-5** |
| **LCA** | **3-4** | **3** | **4** | **3** | **4** |
| **LEL** | **2** | **1-2** | **2** | **2** | **3** |
| **PHA-E** | **5** | **4** | **5** | **4-5** | **5** |
| **PHA-L** | **3** | **3** | **2** | **2-3** | **2** |
| **PNA** | **2** | **3** | **1** | **2** | **2** |
| **PSA** | **4** | **3** | **4** | **3** | **4** |
| **RCA1** | **5** | **4** | **5** | **4-5** | **5** |
| **SBA** | **2** | **2** | **2** | **2** | **1-2** |
| **SJA** | **1** | **1** | **-** | **1** | **-** |
| **STL** | **3** | **2-3** | **2-3** | **2** | **2-3** |
| **UEA I** | **2** | **2** | **2** | **2** | **2** |
| **VVL** | **2** | **3** | **2** | **2** | **2-3** |
| **WFL** | **2** | **3** | **2** | **3** | **2** |
| **WGA** | **4** | **3** | **4** | **3** | **4** |
| **sWGA** | **2** | **2** | **1** | **1-2** | **2** |

***** Binding of biotinylated lectins (10µg/ml) was compared with the level of binding of 5 µg/ml antibody to MHC class-I (standard). Results are from 3 experiments and are expressed as the binding range for each lectin, where - = no detectable binding, 1 = <25%, 2 = 25%-75%, 3 = 75%-125%, 4= 125%-175% and 5 = >175% of the MHC class-I.

Lectins used were: ConA, concanavalin-A; DBA, Dolichus biflorus agglutinin; DSL, Daturum stramonium lectin; ECL, Erythina crystagalli lectin; GSL, Griffonia (Bandeiraea) simplificifolia lectins I, II and isolectin B4; Jacalin; LCA, Lens culinaris agglutinin; LEL, Lycopersicon esculentum (tomato) lectin; PHA-E, Phaseus vulgaris erythroagglutinin; PHE-L, Phaseus vulgaris leucoagglutinin; PNA, peanut agglutinin; PSA, Pisum sativum agglutinin; RCA1, Ricinus communis agglutinin; SBA, Soybean agglutinin; SJA, Sophora japonica agglutinin; STL, Solanum tubersosum (potato) lectin; UEA I, Ulex europaeus agglutin I; VVL, Vicia villosa lectin; WFL, Wisteria floribunda lectin; WGA, Wheat germ agglutinin; sWGA, succinylated wheat germ agglutinin.

**¶** Human endothelial cells were prepared as described (Hillyer P and Male DK (2005) Expression of chemokines on the surface of different human endothelia. Immunol. Cell Biol. **83**, 375-382) and those used were: BMEC, Bone marrow endothelial cells; SVEC, saphenous vein endothelial cells; HUVEC, human umbilical vein endothelial cells; DMVEC, dermal microvascular endothelial cells; LMVEC, lung microvascular endothelial cells.
